# Material Hardship Among Affluent and Poor Households in the United States

**DOI:** 10.1007/s11113-026-10019-1

**Published:** 2026-06-12

**Authors:** John Iceland, Jaehoon Cho

**Affiliations:** Department of Sociology, Pennsylvania State University, University Park, USA

**Keywords:** Hardship, Poverty, Affluence, Decomposition analysis

## Abstract

We examined the correlates of hardship among affluent households and the characteristics of affluent households make them less likely to report hardships than poor ones. Is it income alone that explains differences in the likelihood of hardship, or do other characteristics—such as wealth and education—play important roles? Using 2021 data from the Survey of Income and Program Participation, we examined the prevalence of four types of hardship (bill paying, food, housing, and neighborhood) among poor and affluent households, with affluence defined as household income at least five times the poverty line. The predictors of hardship generally did not significantly differ across poor and affluent households. Wealth and the presence of a disabled household member were consistently linked with hardship among both. Decomposition analyses indicated that the difference in income alone was the most important reason why poor households are more likely to experience bill-paying and food hardships than affluent households, but wealth played the largest role in explaining differences in housing and neighborhood hardships. In decomposition analyses without income and wealth—two proximate determinants of hardship—household type (e.g., presence of a married couple), education, employment status, and race helped explain differences in hardship prevalence across poor and affluent households. Overall, our findings suggest that a variety of characteristics—the most important being income and wealth, depending on the hardship—help explain differences in the prevalence of hardship between poor and affluent households.

**Supplementary Information:**

The online version contains supplementary material available at 10.1007/s11113-026-10019-1.

## Introduction and Background

Millions of Americans experience struggle to meet their basic needs each year. There are a variety of ways to measure economic deprivation, including poverty indicators—such as the official U.S. poverty measure—and measures of specific hardships—such as trouble paying bills (National Research Council, 1995). Research has indicated that there is only a moderate overlap between those counted as poor and those who report a hardship, and the association between the two has weakened over time (Iceland et al., 2021; Iceland & Bauman, 2007; Mayer & Jencks, 1989, 1993). Specifically, many poor households report no hardships, while many nonpoor households, including some affluent ones, report at least one.

There are several reasons for the moderate correlation between income poverty and hardship. First, conceptually they measure different dimensions of well-being. Poverty indicators measure whether households have sufficient income to meet basic needs, such as food, clothing, housing, and other such expenditures, and it is usually measured annually (National Research Council, 1995). In contrast, hardship indicators are designed to be direct measures of the extent to which families experience specific problems, such as food insecurity, difficulty paying bills, or living in substandard housing, often measured as having occurred at any point in the past year (Beverly, 2001; Heflin et al., 2009; Mayer & Jencks, 1989). Households may struggle with these hardships if they experience a change during the year—such as a loss of a member, a job loss, or a decline in earnings—even if the household’s annual income appears to be sufficient to avoid poverty.

In addition, there are several drawbacks to the official poverty measure for determining who is struggling to meet basic needs. Among them, the measure includes only income, though households often have other resources to draw upon, such as wealth. Indeed, previous research has shown that wealth helps mitigate experiences of hardship (Rodems & Pfeffer, 2021). In addition, some people also have health insurance while others do not, and health insurance helps households meet their needs in times of health emergencies. Likewise, some households have high fixed costs—such as high housing payment burdens—while others do not (Deidda, 2015; Park & Seo, 2022; Shamsuddin & Campbell, 2021). Having high housing cost burdens means that such households may have little disposable income to meet unanticipated costs, such as a car or home repair.

The moderate overlap between income and material hardship suggests that economic precarity reaches higher up the income distribution than often assumed (Iceland et al., 2021). Our analysis of hardship among affluent households reveals the extent to which housing costs, medical expenses, wealth constraints, or volatile earnings shape well-being even for families with seemingly comfortable income.

This is important because often it is not just the poor who struggle to meet basic needs, but those up the income ladder whose expenses rival their incomes, or otherwise face an expenses shock, such as a medical emergency. This is especially true in high-cost areas. For instance, according to the MIT living wage calculator, a family of four in Boston needs to earn about $74.12 per hour (working for 2080 hours a year)—or about $154,000 per year—to cover the costs of their family’s basic needs (MIT, 2025). While this might sound high, it speaks to the high cost of housing, childcare, and other expenses in many parts of the country.

Increasing housing costs in many areas has been accompanied by the increase in income precarity (Bania et al., 2009; Dynan et al., 2012). This can occur across the income distribution. In a study of income precarity across socioeconomic groups, Nau and Soener (2019) concluded that “In fact, middle-income working families now have the same level of income precarity as the working poor, and families in the top income quintile continue to have elevated precarity levels.” Finally, identifying why some affluent households struggle while others do not could help both households and policy makers devise better strategies to mitigate hardship. If employment changes are key, this suggests that more attention should be paid to policies that provide short-term income enhancement during spells of unemployment. On the other hand, if having high fixed costs in the form of high rents/mortgages is more important, than making housing more affordable would be more helpful.

In short, the goal of this study is to examine a puzzle that arises from these comparisons of poverty and hardship: the fact that a nontrivial number of apparently affluent household—based on their household income—report material hardships (Iceland et al., 2021). We defined affluent households as those whose incomes are at least five times the poverty line (Iceland, 2021), or about $138,700 for a household of four people in 2021 (U.S. Census Bureau, 2022a). To better understand this puzzle, we seek to answer the following questions in this study:

1. What differentiates affluent households that experience hardship from those that do not?
2. Are the correlates of hardship among affluent households the same as for poor households?
3. To what extent are differences in the prevalence of hardship between poor and affluent households due to differences in the characteristics of these two groups, and which characteristics matter most?

The first question is the most basic, as the answer indicates the determinants of hardship among the affluent. The second is more exploratory, and is meant to draw a potentially sharp contrast between how households with very different incomes still might come to experience hardship. Previous research has shown that poor households experience hardship for well-known reasons, including low income, wealth, and instability (Heflin, 2016; Heflin et al., 2011), and we investigate if the determinants among the affluent are the same. If so, this would indicate that affluent households experience fewer hardships than poor ones simply because they have more income to meet basic needs than poor households or they tend to have other characteristics associated with less disadvantage. The final question more carefully interrogates this issue by examining which characteristics play the most important role in explaining differences in the prevalence of hardship between poor and affluent households.

We examine the role of several specific factors that might explain differences in hardship across these households, including household changes (or “shocks”) that might make households susceptible to hardship, such as a change in household size, a loss of a job, or a decline in income; income alone and non-income resources such as wealth; having a high housing cost burden; and other sociodemographic characteristics that have been shown to be associated with hardship, such as age, education, and race/ethnicity.

We investigate these issues using 2021 data from Survey of Income and Program Participation, which contain detailed information on hardships and other household characteristics. Our analysis includes four summary hardship measures, based on 14 constituent hardship items, representing bill-paying hardship, housing hardship, food hardship, and neighborhood problems. While previous studies have indicated that there is a moderate association between poverty and hardship (Iceland et al., 2021; Iceland & Bauman, 2007; Mayer & Jencks, 1989) and other studies have looked at hardship across households with different living arrangements (Heflin & Patnaik, 2022; Iceland & Cho, 2025; Thomas, 2022), our is the first to look at the experience of hardship among the affluent population in the United States.

## Data and Methods

We used 2021 data from multiple panels of the Survey of Income and Program Participation, a nationally representative household survey conducted in the United States (U.S. Census Bureau 2022b). The SIPP is longitudinal survey, with panels lasting from three to five years. The survey is one of the relatively few to collect information on experiences with different kinds of hardship. The SIPP typically has overlapping panels to reduce the effects of seam bias. We used data from the 2020, 2021, and 2022 SIPP panels and observations that represent household characteristics as measured in the 2021 calendar year.

The SIPP, like many surveys, has seen a decline in eligible households participating in the survey. The response rates for the 2020, 2021, and 2022 panels were 36, 42, and 31%, respectively. The 2020 and 2021 panels also experienced respondent attrition in subsequent waves, which reduced the cumulative response rate to 18% and 23% for the 2021 calendar year, respectively (U.S. Census Bureau, 2023). While nonresponse and attrition potentially produce nonrepresentative samples, the U.S. Census Bureau generates weights to offset nonrandom sample loss, based on respondent characteristics including age, sex, race, Hispanic origin, and state of residence. See U.S. Census 2023 for more details. The Covid pandemic also likely had some impact on hardships faced by families, but it is not clear if it affected the correlates of hardship, which is the focus of our analysis. Overall, high nonresponse and attrition rates are a limitation of using the SIPP, but the SIPP remains one of the premier nationally representative longitudinal surveys that also includes a series of questions about experiences of hardship. Future work should extend this analysis to additional years after the pandemic as those data become available.

The original sample included 487,736 individuals from 17,277 households. For the analysis, we restricted the sample to householders as of December of 2021, resulting in 17,419 cases. The number of cases exceeds the number of households because of changes in household membership during the reference year, including new households being formed. Among the 17,419 cases, 11 had sampling weights equal to zero and were therefore excluded from the analytic sample. Thus, our final sample consisted of 17,408, although most of our analyses focused on just affluent and poor households, of which there were 5929 of the former and 2077 of the latter. We used the SIPP household weighting variable in our analyses.

We examined four types of hardship based on 14 constituent measures included in the SIPP: bill-paying hardship, food hardship, housing hardship, and neighborhood hardship.^1^ We categorized a household as experiencing each of these hardships if they answer affirmatively to a certain number of questions, typically based on how previous studies have measured such hardships (Heflin, 2017; Iceland, 2021; Iceland & Sakamoto, 2022). The hardships were defined as follows:

1. *Bill-paying hardship (one or more)*: at any time in the previous year, the household did not pay the full amount of the utility bill (gas, oil, or electricity) or did not pay the full amount of rent or mortgage.
2. *Food hardship (two or more)*: at any time in the previous year, food did not last (and did not have money for more), could not afford to eat balanced meals, cut or skipped meals due to the lack of money, and ate less than should due to the lack of money.
3. *Housing hardship (one or more)*: for the residence where the respondent lived the longest or spent the most time during the year, in the previous year the following conditions were present for the majority of time: problems with pests (such as rats, mice, or roaches), plumbing problems, cracks or holes in walls, and holes in floor.
4. *Neighborhood hardships (two or more)*: for the residence where the respondent lived the longest or spent the most time during the year, in the previous year the following conditions were present for the majority of time: street noise or heavy traffic problems, trash/litter in the streets or lots, members stayed at home for fear of being unsafe, and the neighborhood was unsafe from crime (where the responses “somewhat unsafe and “very unsafe” were recoded as “unsafe”).

The SIPP includes monthly data on many variables, yielding up to 12 observations per household each year ending in December. The hardship data were collected only in December of each year. To align the temporal ordering of predictors and outcomes, we also used the December monthly response for the non-hardship variables, since the unit of analysis in our study was the household year. This approach allowed us to conceptualize hardship as an outcome of other variables measured over the same calendar year. The 14 hardships indicators and summary indicators are shown in Table 1.

**Table 1:** Prevalence of hardship, 2021. *Source*: 2020–2022 SIPP panels

	Total population	Affluent population	Poor population
Bill-paying hardship (1 or more)	8.5	3.1	19.7
Did not pay utility bill	6.4	2.3	15.4
Did not pay mortgage/rent	5.2	1.5	12.4
Food hardship (2 or more)	9.7	2.3	24.3
Food did not last	11.0	2.9	26.9
Did not eat balanced meals	10.7	3.2	24.5
Skipped meals	5.6	1.0	15.3
Ate less than should	5.6	0.8	16.2
Housing hardship (1 or more)	17.4	12.6	25.5
Insect, pest problems	10.2	6.7	16.7
Plumbing problems	6.7	4.9	10.3
Cracks in wall	6.7	4.1	10.9
Holes in floor	1.6	0.9	3.5
Neighborhood hardship (2 or more)	8.8	5.6	16.7
Noise problems	15.0	11.4	20.0
Trash, litter	9.0	6.2	15.1
Stay at home for fear	9.9	7.4	16.8
Neighborhood is unsafe	5.1	2.6	11.0
N	17,409	5929	2077

Affluent population defined as household income over five times the poverty line

Household-level income was constructed by summing the 12 monthly cash income from earnings and other sources, such as cash welfare and social security payments. We operationalized income at the annual level to ensure consistency with the poverty threshold, which was defined on a yearly basis.

Based on previous studies, we defined affluent households as those with incomes above five times the poverty threshold for that household (Farley, 1996; Iceland, 2021; Sakamoto et al., 2022). For a household of two adults and two children, the threshold for affluence was $138,700 in 2021 (U.S. Census Bureau, 2022a). This threshold would place these households very close to the 80th percentile of household income in that year ($149,100) (U.S. Census Bureau 2022c), which is also close to Reeves’ (2017) definition of the upper middle class. Table 1 shows that affluent households were considerably less likely than poor households to report the four hardships, although a nontrivial share still did.

We incorporated variables that measure changes in the household that could have led to hardship at some point during the previous year. These included: (a) a person was added or subtracted from the household, (b) someone in the household lost a job, and (c) the household suffered a monthly income loss greater than $1000.^2^ Our models also contained variables that measure non-income household resources, including wealth and whether the householder had health insurance, plus whether the household has high fixed housing costs. Household wealth was measured by the total net worth of the household in December of the calendar year. Wealth included all assets (e.g., market value of home, stocks and mutual funds, and IRAs, minus liabilities, such as mortgage, credit card, and medical debt). We used the following dollar amount cutoffs for this variable, informed by the distribution of the wealth variable: zero or less wealth; 1$–$49,999; $50,000–$299,999; $300,000–$999,999; and $1,000,000+. Results were similar when we used wealth quintiles. We measured monthly housing costs with the following categories, informed by the distribution of the variable: no housing expenses; $1–$1000; $1001–$1500; and over $1500. We also ran models with an alternative measure of this variable— the ratio of housing costs (rent or mortgage) to total household income. It had a somewhat weaker relationship with hardship than our main measure, indicating that absolute expenses may matter more for understanding hardship than relative ones, such as the ratio.

We also tested models with additional indicators of non-income benefits, including whether the household received Supplemental Nutritional Assistance Program (SNAP), housing subsidies, energy assistance, the Earned Income Tax Credit, and free or reduced-price school lunches, and the household amount of nutrition assistance for women and children (WIC), since these programs have been shown to reduce hardship (McKernan et al., 2021). We decided to omit these as indicators of non-income resources because these were generally associated with more hardship, indicating that positive selection of struggling household into receipt outweighs the benefits the households might receive.

We also included measures of the sociodemographic composition of the household, including: household living arrangements, defined as married-couple households and cohabiting couples, single female-headed households with children, other families (such as single-parent father households or siblings living together), and non-family households; race/ethnicity of the householder, defined as non-Hispanic White, non-Hispanic Black, non-Hispanic Asian, non-Hispanic other race, and Hispanic; nativity status of the householder (native-born or not); age of the householder, with categories for under 25, 25–34, 35–44, 45-54, 55–64, and 65 and over; education of the householder, with categories for less than high school, high school diploma, some college, or bachelor’s degree or more; employment status of householder, with categories for employed full-time, employed part-time, unemployed, and out of the labor force; an indicator for whether the household was in a nonmetropolitan area; region, with the categories for Northeast, Midwest, South, and West; a child under age 18 is present in the household; and the household had a disabled individual. The descriptive statistics for these variables are shown in Table 2.

**Table 2:** Descriptive statistics. *Source*: 2020–2022 SIPP panels

	Mean (Total)	Mean (Affluent)	Mean (Poor)
Affluent households	35.5		
Poor households	11.6		
Household gained/lost a person	6.7	7.1	5.4
Person in household lost job	9.4	8.2	10.5
Income loss $1000 or more	25.1	33.0	13.1
*Household wealth*			
Zero or Less wealth	11.5	4.5	27.2
$1–$49,999	22.9	7.2	44.0
$50,000–$299,999	26.9	22.9	18.6
$300,000–$999,999	22.2	31.1	7.2
Over $1,000,000	16.5	34.3	3.0
Doesn’t have insurance (householder)	10.0	4.7	17.3
*Housing costs*			
No housing price	30.5	27.0	32.1
1–1000	29.6	14.3	48.5
1001–1500	17.2	19.2	11.2
Over 1500	22.7	39.6	8.3
*Household type*			
Married /cohabiting couple	51.1	69.4	21.6
Female-headed with children	6.8	1.7	15.7
Other family	7.7	5.5	7.1
Non-family household	34.4	23.4	55.6
*Race*			
Non-Hispanic White	65.3	73.5	48.6
Non-Hispanic Black	12.7	8.1	23.3
Non-Hispanic Asian	5.5	7.4	4.2
Non-Hispanic other	14.5	9.2	21.1
Hispanic	2.0	1.9	2.8
Native-born	85.9	87.4	82.7
*Age*			
Under 25	4.1	1.9	8.5
25–34	15.0	14.5	16.2
35–44	16.9	18.6	15.1
45–54	16.5	19.7	14.1
55–64	18.8	21.6	20.0
65 or more	28.7	23.8	26.1
Education			
*Less than high school*	8.4	2.2	19.4
High school	24.6	13.2	34.7
Some college	27.4	21.9	29.3
College or more	39.7	62.8	16.6
*Labor force status*			
Unemployed (omitted)	1.7	0.7	6.4
Full-time employed	48.7	67.2	12.9
Part-time employed	12.8	10.3	16.4
Out of labor force	36.8	21.8	64.2
In nonmetro area	13.6	8.7	16.5
*Region*			
West (omitted)	22.7	26.6	18.3
Midwest	21.4	20.3	18.2
Northeast	17.4	19.5	17.4
South	38.6	33.6	46.1
Children present	26.1	22.8	26.9
Disabled person present	26.6	15.3	43.1
N	17,409	5929	2077

We began the analysis by running logistic models predicting hardship among poor and affluent households and determined whether the coefficients for the predictors described above differed across these two household types. These models answered the first two questions posed: (1) What differentiates affluent households that experience hardship from those that do not? (2) Are the correlates of hardship among affluent households the same as for poor households?

To answer the third research question (To what extent are differences in the prevalence of hardship between poor and affluent households due to differences in the characteristics of these two groups, and which characteristics matter most? ), we conducted a decomposition analysis using a variant of the Blinder-Oaxaca decomposition for linear regression (Blinder, 1973; Oaxaca, 1973) developed by Fairlie (2005) and Bauer and Sinning (2006) for nonlinear regression models. This decomposition method allowed us to estimate the role of group characteristics in explaining differences in the prevalence of hardship among the poor versus affluent households. We used pooled regression coefficients (from a weighted sample that includes households of the two groups) and applied them to means of both groups (Neumark, 2004; Oaxaca & Ransom, 1994). In sensitivity analyses, we found that using different reference group coefficients sometimes affected the statistical significance of the effect of specific endowments in some of the decomposition models—they were more likely to be nonsignificant when using specific reference groups, in part due to smaller sample sizes than in pooled models—but it did not change this study’s general conclusions.

In our main decomposition model, we included an income dummy indicator (i.e., whether the household is poor or affluent) to see the extent to which income alone explained differences in hardship across groups. We also ran decompositions without income and wealth to see the extent to which other variables explained differences in hardship, since income and wealth can be considered proximate determinants of hardship. For example, one of the main reasons education and race might be associated with hardship is that they affect income and wealth, although it is possible that each of these might have effects independent of income and wealth alone (e.g., those with higher education might have greater self-efficacy that helps them avoid hardship than those with lower levels of education—net of income and wealth).

## Results

Table 3 shows logistic results for bill-paying and food hardships, while Table 4 shows results for housing and neighborhood hardships. One of two characteristics that was most consistently associated with hardship across both affluent and poor households was wealth, with higher wealth associated with a lower likelihood of all four hardships. The second characteristic is the presence of a disabled person present in the household, which was consistently associated with a higher likelihood of all four hardships in all but one of the models.

**Table 3:** Logistic regression: bill-paying and food hardships

	Bill-paying hardship				Food hardship			
	Model 1 (affluent)		Model 2 (poor)		Model 1 (affluent)		Model 2 (poor)	
Household gained/lost a person	− 0.309	(0.415)	0.069	(0.304)	0.611+	(0.347)	0.375	(0.301)
Person in household lost job	0.149	(0.313)	− 0.184	(0.273)	− 1.258** ^a^	(0.470)	0.008 ^a^	(0.242)
Income loss $1000 or more	0.577**	(0.208)	0.317	(0.266)	0.596* ^a^	(0.241)	− 0.069 ^a^	(0.227)
*Household wealth*								
Zero or Less Wealth (omitted)								
$1–$49,999	− 1.067** ^a^	(0.356)	− 0.300 + ^a^	(0.162)	− 0.165	(0.450)	− 0.353*	(0.151)
$50,000–$299,999	− 1.680*** ^a^	(0.303)	− 0.614** ^a^	(0.236)	− 0.985*	(0.399)	− 0.705***	(0.202)
$300,000–$999,999	− 2.173***	(0.320)	− 2.099***	(0.475)	− 1.370***	(0.403)	− 1.516***	(0.367)
Over $1,000,000	− 2.902***	(0.399)	− 1.852**	(0.674)	− 2.102***	(0.440)	− 1.168*	(0.537)
Doesn’t have insurance (householder)	0.162	(0.385)	0.402*	(0.200)	0.243	(0.397)	0.166	(0.186)
*Housing costs*								
No housing price (omitted)								
1–1000	0.079	(0.303)	− 0.051	(0.185)	− 0.813* ^a^	(0.332)	0.076 ^a^	(0.153)
1001–1500	− 0.459 ^a^	(0.304)	0.418 ^a^	(0.255)	− 0.658*	(0.309)	− 0.189	(0.254)
Over 1500	− 0.248	(0.278)	0.021	(0.310)	− 0.501+	(0.281)	− 0.359	(0.298)
*Household type*								
Married /cohabiting couple (omitted)								
Female-headed with children	0.833	(0.510)	0.626*	(0.268)	0.664	(0.621)	0.212	(0.264)
Other family	− 0.465	(0.419)	− 0.103	(0.305)	− 0.397	(0.378)	− 0.082	(0.270)
Non-family household	− 0.149	(0.260)	− 0.229	(0.234)	− 0.046	(0.264)	0.048	(0.202)
*Race*								
Non-Hispanic White (omitted)								
Non-Hispanic Black	1.339***	(0.276)	0.751***	(0.188)	0.348	(0.346)	− 0.125	(0.171)
Non-Hispanic Asian	− 0.036	(0.443)	− 1.207*	(0.542)	− 0.208	(0.522)	− 0.582	(0.386)
Non-Hispanic other	0.657*	(0.266)	0.548*	(0.215)	0.111	(0.329)	0.108	(0.202)
Hispanic	1.006*	(0.452)	0.725*	(0.335)	0.626	(0.456)	− 0.269	(0.351)
Native-born	0.278	(0.297)	− 0.225	(0.226)	− 0.487	(0.361)	− 0.480*	(0.204)
*Age*								
Under 25 (omitted)								
25–34	0.456	(0.573)	0.663+	(0.355)	− 0.659	(0.578)	0.541+	(0.309)
35–44	0.561	(0.579)	0.695+	(0.356)	− 0.392	(0.581)	0.188	(0.313)
45–54	1.104+	(0.566)	0.475	(0.369)	0.011	(0.603)	0.199	(0.318)
55–64	0.965+	(0.584)	0.770*	(0.352)	− 0.313	(0.588)	0.185	(0.295)
65 or more	0.110	(0.625)	0.495	(0.357)	− 1.226+	(0.650)	− 0.257	(0.297)
*Education*								
Less than high school (omitted)								
High school	0.169	(0.538)	− 0.001	(0.202)	− 0.352	(0.498)	0.075	(0.176)
Some college	0.084	(0.528)	0.395+	(0.209)	− 0.088	(0.473)	− 0.010	(0.191)
College or more	− 0.005	(0.503)	− 0.089	(0.294)	− 0.505	(0.459)	− 0.466+	(0.268)
*Labor force status*								
Unemployed (omitted)								
Full-time employed	− 0.953	(0.591)	− 0.845**	(0.318)	1.087	(1.332)	− 1.224***	(0.295)
Part-time employed	− 0.527	(0.622)	− 0.440	(0.273)	1.520	(1.346)	− 1.091***	(0.270)
Out of labor force	− 0.799	(0.618)	− 0.899***	(0.248)	1.722 ^a^	(1.339)	− 1.089*** ^a^	(0.229)
In nonmetro area	− 0.667	(0.435)	0.057	(0.193)	− 0.212	(0.364)	0.169	(0.161)
*Region*								
West (omitted)								
Midwest	− 0.251	(0.274)	− 0.203	(0.235)	0.118	(0.312)	0.179	(0.213)
Northeast	0.192	(0.238)	− 0.025	(0.227)	− 1.121** ^a^	(0.371)	− 0.133 ^a^	(0.218)
South	− 0.599*	(0.254)	− 0.410*	(0.194)	− 0.083	(0.243)	− 0.020	(0.167)
Children present	0.099	(0.245)	0.141	(0.282)	− 0.325	(0.335)	− 0.015	(0.262)
Disabled person present	0.295	(0.224)	0.483**	(0.180)	0.676**	(0.217)	0.837***	(0.144)
Constant	− 1.922+	(1.026)	− 1.368*	(0.574)	− 2.388	(1.504)	0.055	(0.503)
Observations	5929		2077		5929		2077	
BIC	10930267.5		13229343.3		8949829.6		15471198.7	

Standard errors in parentheses. * *p* < 0.05 ** *p* < 0.01 *** *p* < 0.001. a = coefficient from the affluent & poor models significantly differ at *p* < 0.05

**Table 4:** Logistic regression: housing and neighborhood hardships

			Housing hardship				Neighborhood hardship			
			Model 1 (affluent)		Model 2 (poor)		Model 1 (affluent)		Model 2 (poor)	
Household gained/lost a person			− 0.083	(0.183)	0.261	(0.265)	− 0.164	(0.190)	− 0.421	(0.283)
Person in household lost job			− 0.237	(0.186)	0.131	(0.242)	− 0.138	(0.175)	− 0.220	(0.249)
Income loss $1000 or more			0.092	(0.105)	0.195	(0.222)	0.121	(0.101)	0.327	(0.215)
*Household wealth*										
Zero or Less wealth (omitted)										
$1–$49,999			0.006	(0.274)	− 0.298*	(0.143)	− 0.678**	(0.247)	− 0.172	(0.145)
$50,000–$299,999			− 0.288	(0.244)	− 0.830***	(0.208)	− 0.417*	(0.205)	− 0.534**	(0.190)
$300,000–$999,999			− 0.320	(0.242)	− 0.812**	(0.270)	− 0.779***	(0.207)	− 0.935**	(0.297)
Over $1,000,000			− 0.477 + ^a^	(0.253)	− 1.601** ^a^	(0.503)	− 1.092***	(0.219)	− 1.323**	(0.457)
Doesn’t have insurance (householder)			0.255	(0.205)	0.137	(0.174)	0.148	(0.198)	− 0.333+	(0.179)
*Housing costs*										
No housing price (omitted)										
1–1000			− 0.084	(0.154)	− 0.188	(0.154)	− 0.056	(0.150)	− 0.278+	(0.150)
1001–1500			− 0.201	(0.147)	− 0.071	(0.223)	− 0.338*	(0.148)	− 0.490*	(0.230)
Over 1500			− 0.098 ^a^	(0.128)	− 0.874** ^a^	(0.294)	− 0.135 ^a^	(0.122)	− 0.859** ^a^	(0.295)
*Household type*										
Married /cohabiting couple (omitted)										
Female-headed with children			0.439	(0.291)	− 0.128	(0.251)	− 0.005	(0.339)	0.221	(0.255)
Other family			0.315+	(0.173)	0.353	(0.258)	0.187	(0.180)	0.356	(0.264)
Non-family household			0.091	(0.119)	0.202	(0.194)	0.327**	(0.108)	0.325+	(0.189)
*Race*										
Non-Hispanic White (omitted)										
Non-Hispanic Black			0.119	(0.188)	0.041	(0.164)	0.525**	(0.161)	0.463**	(0.159)
Non-Hispanic Asian			0.119	(0.200)	0.011	(0.363)	0.247	(0.182)	0.157	(0.313)
Non-Hispanic other			0.270	(0.175)	0.245	(0.181)	0.297+	(0.157)	0.261	(0.190)
Hispanic			0.483+	(0.286)	0.321	(0.314)	− 0.281	(0.301)	0.536+	(0.301)
Native-born			0.142	(0.169)	0.406*	(0.197)	0.186	(0.147)	0.176	(0.196)
*Age*										
Under 25 (omitted)										
25–34			− 0.086	(0.352)	0.403	(0.304)	0.019	(0.307)	− 0.076	(0.291)
35–44			− 0.237	(0.360)	0.352	(0.308)	− 0.119	(0.315)	0.020	(0.296)
45–54			− 0.175	(0.355)	0.315	(0.312)	− 0.080	(0.311)	− 0.051	(0.290)
55–64			− 0.414	(0.354)	0.449	(0.295)	− 0.470	(0.313)	0.166	(0.276)
65 or more			− 0.437	(0.359)	0.163	(0.298)	− 0.752*	(0.321)	− 0.238	(0.270)
*Education*										
Less than high school (omitted)										
High school			0.235	(0.304)	− 0.184	(0.168)	0.067	(0.270)	− 0.178	(0.171)
Some college			0.065	(0.298)	0.033	(0.176)	− 0.125	(0.267)	0.043	(0.178)
BA or more			0.069	(0.291)	− 0.030	(0.221)	− 0.245	(0.260)	0.281	(0.217)
*Labor force status*										
Unemployed (omitted)										
Full-time employed			− 0.428	(0.462)	− 0.175	(0.299)	− 0.211	(0.491)	− 0.638*	(0.284)
Part-time employed			− 0.449	(0.477)	− 0.444	(0.276)	− 0.186	(0.507)	− 0.621*	(0.268)
Out of labor force			− 0.293	(0.471)	− 0.211	(0.245)	− 0.057	(0.500)	− 0.914***	(0.231)
In nonmetro area			− 0.135	(0.160)	0.084	(0.152)	− 0.103	(0.161)	− 0.023	(0.154)
*Region*										
West (omitted)										
Midwest			− 0.033	(0.142)	− 0.195	(0.203)	− 0.684***	(0.144)	− 0.457*	(0.197)
Northeast			0.352** ^a^	(0.134)	− 0.225 ^a^	(0.196)	− 0.009	(0.124)	− 0.119	(0.194)
South			− 0.005	(0.115)	− 0.067	(0.161)	− 0.530***	(0.110)	− 0.321*	(0.160)
Children present			0.025	(0.136)	0.294	(0.245)	− 0.305*	(0.137)	0.232	(0.250)
Disabled person present			0.437***	(0.115)	0.734***	(0.139)	0.302* ^a^	(0.119)	0.713*** ^a^	(0.139)
Constant			− 1.335*	(0.665)	− 1.530**	(0.485)	− 0.399	(0.684)	− 0.415	(0.472)
Observations			5929		2077		5929		2077	
BIC			35166299.1		16435659.3		36364436.7		16551145.1	

Standard errors in parentheses. * *p* < 0.05 ** *p* < 0.01 *** *p* < 0.001. a =coefficient from the affluent & poor models significantly differ at *p* < 0.05

The associations between other characteristics and hardship were more variable. Among poor households, those with a householder who is employed full-time or out of the labor force were less likely to experience three of the four hardships than those who were unemployed, but employment status was nonsignificant among affluent households. Among affluent households, an income loss of $1000 was associated with a greater likelihood of bill-paying and food hardship, although not with housing or neighborhood hardships. African Americans were more likely to experience two of the four hardships (bill-paying and neighborhood hardships) among both poor and affluent households, but they were not more likely to experience the other two hardships (food and housing hardships).

The coefficients for yet other variables were only occasionally significant (e.g., household type, income loss, person in the household lost a job, and region)—net of the other variables in the models, making it more difficult to draw strong conclusions about them. Notably, there were relatively few statistically significant differences in the coefficients between poor and affluent households, and not in a consistent pattern across the four hardships.

Table 5 shows results from the decomposition analysis. Since the association between most of the variables and hardship did not significantly differ across poor and affluent households, it was useful to see which characteristics were most important in explaining the difference in the likelihood of hardship across these two groups. In this decomposition analysis we also included the income dummy variable to see if the difference in poor/affluent status itself explained the difference in hardship. That is, are affluent households less likely to experience hardship simply because they have a lot more income to meet basic needs than poor households?

**Table 5:** Decompositions of differences in hardship, by type of hardship and poverty/affluence status

	Bill-paying hardship	Food hardship	Housing hardship	Neighborhood hardship
Affluent % experiencing hardship	0.20	0.24	0.26	0.26
Poor % experiencing hardship	0.03	0.02	0.13	0.14
Difference	0.16	0.22	0.13	0.12
Total explained by each factor				
Household gained/lost a person	0%	− 1%	0%	1%
Person in household lost job	0%	− 1%	0%	− 1%
Income loss $1000 or more	− 12%**	− 5%	− 3%	− 5%
Income	64%***	73%***	21%	0%
Total household net worth	28%***	19%***	38%***	51%***
Doesn’t have insurance	1%	0%	2%	− 2%
Housing costs	− 1%	2%	4%	8%
Household type	5%*	1%	6%	15%v
Race	7%***	0%	4%	11%***
Native-born	0%	0%	− 1%*	− 1%
Age	0%	0%	− 2%	− 3%
Education	1%	3%*	3%	5%
Labor force status	2%	5%*	8%	3%
Region/metro status	0%	0%	0%	− 3%*
Children present	1%	0%	1%	− 1%
Disabled person present	2%*	4%***	19%***	18%***

* *p* < 0.05 ** *p* < 0.01 *** *p* < 0.001

The results indicated that income played a key role in explaining the difference in the prevalence of two of the four hardships across poor and affluent households. Specifically, income differences explained 64–73% of the difference in bill-paying and food hardship. While it seemed to explain a substantively important portion of the difference in housing hardship (21%), this was not statistically significant, and income explained little of the difference in neighborhood problems. Instead, wealth played the largest role in explaining differences in housing and neighborhood hardships—38 to 51%—and was also important in explaining differences in bill-paying and food hardship (19–28%). The only other variable that was significant in all four hardship models was the presence of a disabled person in the household, which explained from 2% to 19% of the difference in hardship. In other words, one reason poor household were more likely to report hardships—net of income, wealth, and other characteristics—was that they were more likely to have a disabled person present, which was strongly correlated with hardship. The only variable that is significant in at least two of the hardship decompositions was race, which helped explain differences in bill-paying and neighborhood hardships. As shown in Tables 3 and 4, African Americans were more likely to experience these two hardships than White households, and African Americans were over-represented among the poor population.

Results from decomposition analyses omitting income and wealth—two proximate determinants of hardship— indicated that several other variables helped explain differences in hardship across poor and affluent households mainly through their effect on income and wealth (see Table S1 in the Online Supplement). These included household type—married couple households were considerably more prevalent among affluent households than poor households. Education, labor force status, and race were important in most models, indicating that affluent households were less likely to report hardships because they tended to have higher levels of education, were more likely to be employed, and were more likely to have a White or Asian householder than a Black or Hispanic householder than poor households. All these characteristics were associated with hardship, especially in pooled models without wealth (see Table S2 in the Online Supplement).

## Discussion and Conclusion

We examine why some affluent households experience material hardship, and what characteristics of affluent households make them less likely to report hardship than poor households. Is it income alone that distinguishes affluent households from poor ones, or are other factors such as wealth, having health insurance, or other demographic characteristics such as age and race? Using 2021 data from the Survey of Income and Program Participation, we examined reports of four summary hardship measures, based on 14 constituent hardship items, representing bill-paying, housing, food, and neighborhood hardships. We defined affluent households as those whose incomes are at least five times the poverty line (Iceland, 2021), or about $138,700 for a household of four people in 2021 (U.S. Census Bureau, 2022a). We ran logistic regression models predicting hardship among poor and affluent households to see if the correlates of hardship vary across these households, and then conducted demographic decomposition models to gauge which characteristics explained differences in the likelihood of hardship across them.

We find that the two characteristics most consistently associated with hardship are wealth—higher wealth is linked to a lower likelihood of all four hardships among both poor and affluent households—and the presence of a disabled household member, which is consistently linked to a higher likelihood of experiencing all four hardships. The finding that disability is strongly associated with hardship is consistent with past studies on this issue, as households with a disabled member may have lower earnings if the disabled individual has a limited ability to work, and have higher expenses for therapy, transportation, health care, and other items related to adapting the home environment and assistive technology. Health insurance often does not cover all these expenses (Parish et al., 2009; She & Livermore, 2007).

On the whole, there are relatively few statistically significant differences in the coefficients predicting hardship across poor and affluent households, and not in a consistent pattern across the four hardships. This suggests that we cannot not with confidence conclude that the factors that produce, or at least that are associated with, hardship among affluent households are altogether different than those associated with hardship among poor ones.

The decomposition analysis indicates that the difference in income alone is the most important reason why poor households are more likely to experience bill-paying and food hardship than affluent ones (explaining between 64% and 73% of the difference), but that wealth plays the largest role in explaining differences in housing and neighborhood hardships (between 38% and 51% of the difference). This is consistent with the notion that food and bill-paying hardships are more affected by short-term income, while housing hardship and neighborhood problems are more strongly associated with longer-term income (Iceland et al., 2021 b; Iceland & Bauman, 2007) and, as shown in this analysis—*wealth*. Wealth has been found to mitigate hardship by insulating households from disruptive events and more generally serves as a private safety net (Rodems & Pfeffer, 2021). The next most consistent factor explaining differences in the likelihood of hardship was the presence of a disabled household member, which was more common among poor than affluent households.

In decomposition analyses that omitted income and wealth, which are two proximate causes of hardship, we find that household type played a prominent role in explaining differences in the likelihood of hardship across poor and affluent households, along with education, labor force status, and race. Affluent households are more likely to have a married couple, higher levels of education, are more likely to be employed, and more likely to have White or Asian householders than poor households. All these characteristics are associated with lower levels of hardship.

One of the implications of our findings is that poor and affluent households experience hardship for many of the same reasons. Affluent households are less likely to experience hardship than poor ones because of a variety of protective factors, not the least being having more income and wealth. Nonetheless, a second implication of our analysis is that a nontrivial number of affluent households still experience hardship, even as they seemingly have sufficient income to insulate them from such hardship. This suggests that many affluent households have expenses that closely match their income. In high-cost areas, for instance, they may spend a lot on childcare and housing expenses—perhaps in similar proportions as poor households—but nonetheless their elevated expenditures produce precarity (Bania et al., 2009; Dynan et al., 2012; Nau & Soener, 2019).

Finally, the results raise questions about the measurement of poverty and deprivation. Is it more informative to measure poverty as the lack of resources to meet basic needs over the course of a year? Or whether a household experiences a distinct hardship, such as missing a bill due to a lack of money in a given month? Should we be concerned about households that have relatively high incomes but still experience hardship? Or conversely, about households that report low incomes but never report any material hardships? Which of these households is better off? Future research, including ethnographic work, should delve into why a non-trivial number of affluent households experience these hardships, while so many seemingly poor households do not.

## Supplementary Information

Below is the link to the electronic supplementary material.

Supplementary Material 1

## Data Availability

No datasets were generated or analysed during the current study.

## Footnotes

1 Principal components factor analysis indicated that these 14 indicators represented four factors of hardship.

2 The $1000 amount is similar to the threshold used by Heflin (2016) ($750) when adjusted for inflation between 2009 and 2021.
