# Supplementary material for: Material Hardship Among Affluent and Poor Households in the United States
Source: Popul Res Policy Rev. 2026 Jun 12;45(4):35. doi: 10.1007/s11113-026-10019-1 (PMC13263291; doi:10.1007/s11113-026-10019-1)
Supplement: Supplementary file 1 — Supplementary Material 1 [file 11113_2026_10019_MOESM1_ESM.docx]

# ONLINE SUPPLEMENT

**Material Hardship among Affluent and Poor Households in the United States**

Table S1. *Decompositions of Differences in Hardship, by Type of Hardship and Poverty/Affluence Status*

|  | Bill-Paying  Hardship | | Food  Hardship | | Housing  Hardship | | Neighborhood Hardship | |
| --- | --- | --- | --- | --- | --- | --- | --- | --- |
| Affluent % experiencing hardship | 0.2 |  | 0.24 |  | 0.26 |  | 0.26 |  |
| Poor % experiencing hardship | 0.03 |  | 0.02 |  | 0.13 |  | 0.14 |  |
| Difference | 0.17 |  | 0.22 |  | 0.13 |  | 0.12 |  |
| Total explained by each factor | 75% |  | 76% |  | 81% |  | 85% |  |
| Household gained/lost a person | 0% |  | -1% |  | 0% |  | 0% |  |
| Person in household lost job | 0% |  | 0% |  | 0% |  | -1% |  |
| Income loss $1000 or more | -4% |  | 0% |  | -2% |  | -4% |  |
| Doesn't have insurance (householder) | 5% | ** | 3% | * | 5% | * | -1% |  |
| Housing Costs | 8% | * | 14% | *** | 10% |  | 13% | * |
| Household type | 19% | *** | 14% | *** | 16% | *** | 26% | *** |
| Race | 15% | *** | 5% | ** | 8% | ** | 16% | *** |
| Native-born | 1% |  | 0% |  | -1% |  | -1% |  |
| Age | -1% |  | -1% |  | -2% |  | -2% |  |
| Education | 9% | ** | 15% | *** | 11% | * | 14% | ** |
| Labor force status | 14% | *** | 20% | *** | 17% | ** | 8% |  |
| Region/metro status | -1% |  | 0% |  | 1% |  | -3% | * |
| Children present | 2% |  | 0% |  | 1% |  | -1% | * |
| Disabled person present | 5% | *** | 8% | *** | 23% | *** | 22% | *** |
| * p<0.05 ** p<0.01 *** p<0.001 | | | | |  | |  | |

*Table S2. Logistic Regressions without Wealth*

|  | Bill-Paying  Hardship | | Food  Hardship | | Housing  Hardship | | Neighborhood  Hardship | |
| --- | --- | --- | --- | --- | --- | --- | --- | --- |
| Household gained/lost a person | -0.010 | (0.225) | 0.459+ | (0.237) | 0.036 | (0.145) | -0.205 | (0.162) |
| Person in household lost job | 0.005 | (0.198) | -0.329 | (0.215) | -0.096 | (0.141) | -0.178 | (0.140) |
| Income loss $1000 or more | 0.184 | (0.151) | -0.044 | (0.168) | 0.059 | (0.093) | 0.125 | (0.089) |
| Doesn't have insurance (householder) | 0.610*** | (0.178) | 0.393* | (0.159) | 0.266* | (0.127) | -0.038 | (0.133) |
| Housing Costs (decile) |  |  |  |  |  |  |  |  |
| No housing price (omitted) |  |  |  |  |  |  |  |  |
| 1-1000 | 0.328* | (0.150) | 0.332** | (0.125) | 0.067 | (0.096) | 0.072 | (0.098) |
| 1001-1500 | 0.246 | (0.182) | -0.170 | (0.186) | -0.098 | (0.118) | -0.230+ | (0.117) |
| Over 1500 | -0.085 | (0.189) | -0.403* | (0.188) | -0.140 | (0.108) | -0.146 | (0.100) |
| Household Type |  |  |  |  |  |  |  |  |
| Married /cohabiting couple (omitted) |  |  |  |  |  |  |  |  |
| Female-headed with children | 1.106*** | (0.206) | 0.700** | (0.226) | 0.264 | (0.169) | 0.464** | (0.166) |
| Other family | 0.113 | (0.224) | 0.182 | (0.208) | 0.380** | (0.139) | 0.328* | (0.141) |
| Non-family household | 0.410** | (0.151) | 0.695*** | (0.142) | 0.292*** | (0.088) | 0.450*** | (0.087) |
| Race |  |  |  |  |  |  |  |  |
| Non-Hispanic White (omitted) |  |  |  |  |  |  |  |  |
| Non-Hispanic Black | 1.203*** | (0.160) | 0.260 | (0.158) | 0.187 | (0.121) | 0.592*** | (0.113) |
| Non-Hispanic Asian | -0.600+ | (0.334) | -0.395 | (0.310) | 0.107 | (0.171) | 0.192 | (0.157) |
| Non-Hispanic other | 0.918*** | (0.158) | 0.469** | (0.167) | 0.331** | (0.125) | 0.337** | (0.121) |
| Hispanic | 1.073*** | (0.265) | 0.218 | (0.289) | 0.480* | (0.205) | 0.195 | (0.202) |
| Native-born | -0.193 | (0.171) | -0.603*** | (0.183) | 0.204 | (0.128) | 0.155 | (0.116) |
| Age |  |  |  |  |  |  |  |  |
| Under 25 (omitted) |  |  |  |  |  |  |  |  |
| 25-34 | 0.507+ | (0.302) | 0.143 | (0.276) | 0.160 | (0.221) | 0.088 | (0.203) |
| 35-44 | 0.438 | (0.301) | -0.175 | (0.280) | -0.037 | (0.226) | -0.079 | (0.206) |
| 45-54 | 0.396 | (0.303) | -0.181 | (0.287) | -0.055 | (0.220) | -0.167 | (0.205) |
| 55-64 | 0.313 | (0.294) | -0.415 | (0.259) | -0.217 | (0.213) | -0.425* | (0.198) |
| 65 or more | -0.356 | (0.303) | -1.208*** | (0.265) | -0.444* | (0.213) | -0.799*** | (0.196) |
| Education |  |  |  |  |  |  |  |  |
| Less than high school (omitted) |  |  |  |  |  |  |  |  |
| High school | -0.143 | (0.189) | -0.129 | (0.162) | -0.177 | (0.139) | -0.201 | (0.139) |
| Some college | 0.030 | (0.191) | -0.272 | (0.170) | -0.214 | (0.139) | -0.201 | (0.139) |
| College or more | -0.564** | (0.210) | -0.992*** | (0.194) | -0.312* | (0.140) | -0.378** | (0.141) |
| Labor force status |  |  |  |  |  |  |  |  |
| Unemployed (omitted) |  |  |  |  |  |  |  |  |
| Full-time employed | -1.412*** | (0.240) | -1.979*** | (0.237) | -0.494* | (0.214) | -0.701*** | (0.206) |
| Part-time employed | -0.674** | (0.249) | -1.217*** | (0.254) | -0.514* | (0.226) | -0.652** | (0.220) |
| Out of labor force | -0.852*** | (0.230) | -0.955*** | (0.220) | -0.219 | (0.211) | -0.670** | (0.204) |
| In nonmetro area | -0.021 | (0.166) | 0.172 | (0.142) | 0.036 | (0.106) | 0.023 | (0.108) |
| Region |  |  |  |  |  |  |  |  |
| West (omitted) |  |  |  |  |  |  |  |  |
| Midwest | -0.115 | (0.176) | 0.180 | (0.173) | -0.051 | (0.116) | -0.541*** | (0.114) |
| Northeast | 0.176 | (0.166) | -0.292 | (0.180) | 0.224* | (0.111) | 0.010 | (0.105) |
| South | -0.338* | (0.143) | 0.026 | (0.137) | 0.039 | (0.092) | -0.390*** | (0.088) |
| Children present | 0.363* | (0.183) | 0.203 | (0.201) | 0.127 | (0.117) | -0.227+ | (0.117) |
| Disabled person present | 0.638*** | (0.133) | 1.000*** | (0.112) | 0.670*** | (0.082) | 0.576*** | (0.084) |
| Constant | -2.481*** | (0.465) | -0.706 | (0.450) | -1.575*** | (0.347) | -0.643+ | (0.332) |
| Observations | 8006 |  | 8006 |  | 8006 |  | 8006 |  |
| BIC | 26210324.740 |  | 26357433.103 |  | 52357146.685 |  | 54047070.882 |  |
| Standard errors in parentheses: * p<0.05 ** p<0.01 *** p<0.001 | | | | | | | | |
